# Leveraging diverse cellular stress patterns for predicting clinical outcomes and therapeutic responses in patients with multiple myeloma

**DOI:** 10.1111/jcmm.70054

**Published:** 2024-09-08

**Authors:** Jiaxuan Xu, Xiaoqing Dong, Jiahui Dong, Yue Peng, Mengying Xing, Lanxin Chen, Quan Zhao, Bing Chen

**Affiliations:** ^1^ Department of Hematology Nanjing Drum Tower Hospital, Affiliated Hospital of Medical School, China‐Australia Institute of Translational Medicine, School of Life Sciences, Nanjing University Nanjing China

**Keywords:** cellular stress index, multiple myeloma, nomogram model, therapeutic sensitivity, tumour microenvironment

## Abstract

Tumour microenvironment harbours diverse stress factors that affect the progression of multiple myeloma (MM), and the survival of MM cells heavily relies on crucial stress pathways. However, the impact of cellular stress on clinical prognosis of MM patients remains largely unknown. This study aimed to provide a cell stress‐related model for survival and treatment prediction in MM. We incorporated five cell stress patterns including heat, oxidative, hypoxic, genotoxic, and endoplasmic reticulum stresses, to develop a comprehensive cellular stress index (CSI). Then we systematically analysed the effects of CSI on survival outcomes, clinical characteristics, immune microenvironment, and treatment sensitivity in MM. Molecular subtypes were identified using consensus clustering analysis based on CSI gene profiles. Moreover, a prognostic nomogram incorporating CSI was constructed and validated to aid in personalised risk stratification. After screening from five stress models, a CSI signature containing nine genes was established by Cox regression analyses and validated in three independent datasets. High CSI was significantly correlated with cell division pathways and poor clinical prognosis. Two distinct MM subtypes were identified through unsupervised clustering, showing significant differences in prognostic outcomes. The nomogram that combined CSI with clinical features exhibited good predictive performances in both training and validation cohorts. Meanwhile, CSI was closely associated with immune cell infiltration level and immune checkpoint gene expression. Therapeutically, patients with high CSI were more sensitive to bortezomib and antimitotic agents, while their response to immunotherapy was less favourable. Furthermore, in vitro experiments using cell lines and clinical samples verified the expression and function of key genes from CSI. The CSI signature could be a clinically applicable indicator of disease evaluation, demonstrating potential in predicting prognosis and guiding therapy for patients with MM.

## 1 INTRODUCTION

Multiple myeloma (MM) is a haematological malignancy characterised by clonal infiltration of abnormal plasma cells in the bone marrow.^1^ The incidence and mortality rates of MM rank second only to non‐Hodgkin lymphoma among haematological tumours.^2^ The implementation of autologous stem cell transplantation, immunomodulatory drugs, proteasome inhibitors, and anti‐CD38 monoclonal antibodies has improved patient outcomes.^3^ However, most MM patients are at risk of progression, relapse and drug resistance, and will eventually face the threat of death from disease itself or associated complications. Currently, there is still a lack of biomarker genes with sufficient specificity for guiding prognostic prediction or therapeutic decision‐making in MM.^4^, ^5^ It is imperative to identify novel molecular features and establish practical clinical models for MM personalised therapy.

Cellular stress is a defence response of cells to enhance their adaptive and survival ability under the stimulation of various environmental stressors. Human cells activate relevant mechanisms that support cell functions in response to stress conditions, thereby maintaining microenvironmental homeostasis.^6^ We categorise the primary patterns of cellular stress into five distinct types: heat stress, oxidative stress, hypoxic stress, genotoxic stress and endoplasmic reticulum (ER) stress. Heat stress is characterised by the activation of heat stress proteins (HSPs) in response to an increasing environmental temperature. As the most highly conserved HSPs, chaperones play a pivotal role in maintaining important cellular structures and functions under stressful conditions.^7^ Oxidative stress arises from the excessive accumulation or inadequate elimination of reactive oxygen species (ROS), resulting in damage to DNA, proteins, or lipids and ultimately promoting malignant tumour behaviours.^8^, ^9^ Hypoxic stress is regarded as a stress situation in which a specific tissue is supplied with less oxygen than required. The most crucial regulator of hypoxic responses is hypoxia‐inducible factor (HIF), which exerts anti‐apoptotic effects and facilitates drug resistance in various cancers.^10^, ^11^ Genotoxic stress is caused by DNA damage response (DDR) that is essential for preserving genome integrity and stability. The complex interaction of DDRs leads to the induction of DNA repair, autophagy, necrosis and senescence to determine cell survival or death.^12^ ER stress is triggered by exposure to intrinsic and extrinsic factors that alter protein homeostasis and disturb ER protein‐folding capacity. Tumour cells will restore ER proteostasis through the unfolded protein response (UPR), which confers greater malignant potential on their growth.^13^, ^14^ The ultimate fate of stressed cells is determined by the interplay between these stress responses, and a thorough understanding of cell stress landscape will facilitate the discovery of innovative therapeutic targets.^15^

To date, few studies have provided a comprehensive overview of cellular stress patterns in cancers, compared with focusing solely on independent stress patterns. The detailed associations between cell stress and MM prognosis remain largely unexplored. Given the crucial role of cell stress in tumour survival, it is urgently required to demonstrate the prognostic and therapeutic value of cellular stress‐related components in patients with MM. In the present study, we developed and validated an integrated cellular stress index (CSI) to predict survival outcomes for MM patients. We also investigated the influence of CSI on clinical features, biological functions, immune status, and drug sensitivity in MM. A predictive nomogram incorporating CSI was established to further aid individualised prognosis assessment and effective risk stratification.

## 2 MATERIALS AND METHODS

### 2.1 Data source

Gene expression profiles and clinicopathological data of patients were retrieved from the MM datasets available on the Gene Expression Omnibus (GEO) database (https://www.ncbi.nlm.nih.gov/geo/) and TCGA MMRF‐COMMPASS project (https://portal.gdc.cancer.gov/projects/MMRF‐COMMPASS). Expression values for each gene were calculated through the robust multi‐array average (RMA) algorithm, and subsequently log2‐transformed to obtain relative mRNA expression levels. The GSE24080 dataset served as the training cohort for index construction and nomogram development, while GSE136337, GSE57317, and MMRF‐COMMPASS datasets were used as validation cohorts. In addition, GSE39754 and GSE6477 datasets were employed for comparing the expression levels of CSI genes between normal tissues and MM samples.

CSI‐related genes encompass the key regulatory genes involved in the aforementioned five cell stress patterns. These genes were obtained from manual collection, previous studies, GeneCards (https://www.genecards.org/), and Molecular signatures Database (https://www.gsea‐msigdb.org/gsea/msigdb/).^16^, ^17^, ^18^, ^19^, ^20^ In total, 60 heat stress‐related genes, 225 oxidative stress‐related genes, 200 hypoxic stress‐related genes, 233 genotoxic stress (DDR)‐related genes and 258 ER stress‐related genes were collected into formal analyses.

### 2.2 Construction of different cellular stress‐related risk score

To generate a prognostic index in each cell stress pattern, univariate Cox regression analysis was performed to identify prognosis‐associated genes (*p* < 0.05) in both GSE24080 and GSE136337 datasets. The intersection of two identified gene sets were further screened using the least absolute shrinkage and selection operator (LASSO) Cox regression algorithm in GSE24080. The penalty parameter λ in LASSO regression was determined using a 10‐fold cross‐validation via minimum criteria. A risk index for each patient was then calculated by multiplying gene expression values with their corresponding regression coefficients. Correlations of five cell stress‐related indexes were visualised using ‘circlize’ R package.

### 2.3 Establishment and assessment of the CSI signature

The genes in five cellular stress‐related models were then taken together to construct a comprehensive CSI for prognosis prediction. LASSO regression analysis was performed to narrow the range of candidates, followed by multivariate Cox analysis to establish the final risk model. The CSI for each individual was derived according to gene expression values and their regression coefficients in multivariate analysis. Patients were stratified into two groups based on the median CSI, and survival probabilities were compared between CSI categories in training and validation cohorts.

Principal component analysis (PCA) was conducted to visualise clustering differences using ‘stats’ package. The ‘pRRophetic’ package was utilised to predict chemotherapeutic sensitivity by estimating the half‐maximal inhibitory concentration (IC50) value for each sample.^21^ Clinical characteristics, including age, sex, bone marrow plasma cells (BMPC, %), lactate dehydrogenase (LDH, U/L), albumin (ALB, g/dL), β2‐microglobulin (BMG, mg/L), haemoglobin (g/dL), creatinine (mg/dL), cytogenetic abnormalities, International Staging System (ISS) stage and survival status, were collected and compared between high and low CSI patients.

### 2.4 Functional enrichment analysis

Identification of differential gene expression was carried out using  ‘limma’ package. The ‘clusterProfiler’ package was utilised to investigate the significantly enriched biological functions of differentially expressed genes in terms of gene ontology (GO). To further validate the biological mechanisms between high and low CSI groups, C5 GO gene sets were downloaded from Molecular signatures Database for gene set enrichment analysis (GSEA). The screening criteria for statistically significant functions were defined as both *p* value and false discovery rate (FDR) below 0.01.

### 2.5 Unsupervised clustering of CSI‐related genes

Unsupervised consensus clustering was performed using R package ‘ConsensusClusterPlus’ in four independent datasets. MM samples were clustered into k classifications with k from 2 to 9, and the optimal number of clusters was evaluated using cumulative distribution function curves and consensus matrices. The relationships between clusters and other features were visualised using Sankey diagrams by R package ‘ggalluvial’.

### 2.6 Development and validation of the nomogram

In the training cohort GSE24080, independent prognostic factors were identified using univariate and multivariate Cox regression analyses. The package ‘regplot’ was employed to generate a nomogram that predicts 2‐, 3‐ and 5‐year overall survival (OS) probabilities. We assessed the predictive accuracy of the nomogram by using calibration plots. Specifically, calibration curves were constructed based on 1000 bootstrap resamples to estimate the degree of deviation between observed and predicted survival probabilities. The discriminative performance of the nomogram model was evaluated using receiver operating characteristic (ROC) curves. The prognostic capacity of the nomogram was compared with other features using the area under the curve (AUC) values in both the training and validation cohorts. Decision curve analysis (DCA) was employed to facilitate the clinical applicability of the nomogram by demonstrating its potential net benefits. Patients were divided into high and low risk groups according to the median score calculated by the nomogram, and Kaplan–Meier curves were plotted based on this nomogram‐based stratification.

### 2.7 Immune microenvironment analysis

Relative infiltration levels of immune cells in the MM microenvironment were characterised by single sample gene set enrichment analysis (ssGSEA). We assessed the associations of each CSI gene with immune cell types by computing Spearman rank correlation coefficients between gene mRNA levels and ssGSEA scores of immune cell gene sets. To further investigate the immune cell infiltration in MM, we utilised the ESTIMATE algorithm to derive immune scores, stromal scores, and tumour purity.^22^ Tumour immune dysfunction and exclusion (TIDE) algorithm was applied to predict immune evasion capacity and immunotherapy responsiveness.^23^ Moreover, we conducted a comparative analysis of the expression profiles of immune checkpoint genes between CSI groups.

### 2.8 Cell lines and patient samples

MM cell lines, including ARP‐1, CAG, LP‐1, NCI‐H929, RPMI‐8226, and U266, were purchased from the National Collection of Authenticated Cell Cultures (Shanghai, China). All cells were cultured in RPMI‐1640 medium (Gibco, USA) supplemented with 10% foetal bovine serum (Biological Industries, Israel) and 1% penicillin–streptomycin (Beyotime Biotechnology, Shanghai, China). The cell culture was conducted in a humidified incubator at 37°C with a 5% CO_2_ atmosphere. Clinical specimens consisted of bone marrow samples from five patients with MM and peripheral blood samples from three healthy individuals. The collection of patient samples was authorised by the Medical Ethics Committee of Nanjing Drum Tower Hospital.

### 2.9 Quantitative real‐time PCR (qRT‐PCR)

Total RNA was extracted using TRIzol reagent (Invitrogen, USA) and reverse transcribed to cDNA using HiScript II Q RT SuperMix (Vazyme, China). Next, qRT‐PCR was conducted using AceQ qPCR SYBR Green Master Mix (Vazyme, China) on a StepOnePlus real‐time PCR system (Applied Biosystems, USA). Relative mRNA level was quantified using the expression of GAPDH as an internal control through the 2^−ΔΔCT^ method. The PCR primer sequences are provided in Table S1.

### 2.10 Construction of stably transfected cell lines

To establish stable overexpression cell lines, the coding sequence of ALDH2 was cloned into a pCDH‐puro vector. On the other hand, the pLKO.1‐puro vector was utilised to achieve stable ENO1‐knockdown cell lines by incorporating ENO1 targeting short‐hairpin RNAs. Lentiviruses were produced through co‐transfection of the lentiviral expression plasmids along with packaging plasmids into HEK293T cells using the Lipofectamine 3000 transfection reagent (Invitrogen, USA). Supernatants were harvested after 48 h and used for infecting MM cells. Positive cells with stable transfection were selected using 1 μg/mL puromycin.

### 2.11 Cell viability assay

CCK‐8 assay was conducted to assess the cell viability after stable transfection. Cells were planted into a 96‐well plate with a volume of 100 μL at a concentration of 5 × 10^3^ cells per well. Then, 10 μL of 1 × CCK‐8 solution (Vazyme Biotech, Nanjing, China) was added and incubated for an additional 2 h at 37°C. Optical density was detected at a wavelength of 450 nm using a Safire microplate reader.

### 2.12 Statistical analysis

All statistical analyses in the present study were performed using R software (version 4.1.1). Distribution of continuous variables was checked for normality using the Shapiro–Wilk test. Comparison of normally distributed data between two groups were analysed using Student's *t* test, otherwise Wilcoxon rank‐sum test was employed. One‐way analysis of variance (ANOVA) or Kruskal‐Wallis test was adopted for multiple group comparisons. Chi‐squared test was applied to compare categorical variables. Survival analyses were carried out using the Kaplan–Meier method and Log‐rank test. Statistical significance was defined by a two‐tailed *p* value less than 0.05. The flow chart of this study is shown in Figure S1.

## 3 RESULTS

### 3.1 Development of five cell stress‐related risk models

**FIGURE 1:**
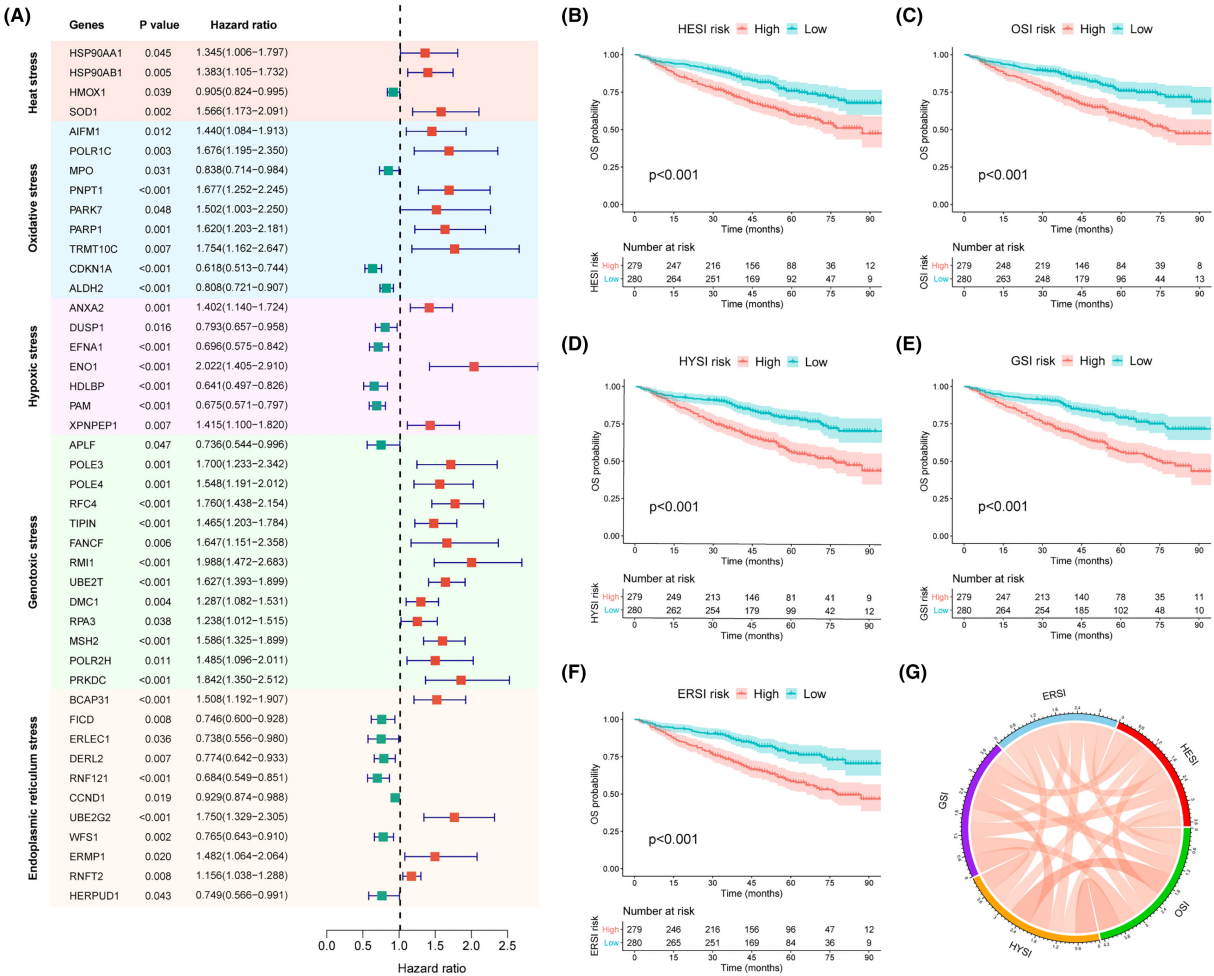
Construction of five cellular stress‐related risk models. (A) An integrated forest plot depicting the prognostic effects of 44 cellular stress‐related genes from five cell stress patterns. HMOX1 belongs to both HESI and HYSI; SOD1 belongs to both HESI and OSI; AIFM1 and PARK7 belong to both OSI and ERSI; PARP1 belongs to both OSI and GSI; CDKN1A belongs to both OSI and HYSI. (B‐F) Kaplan–Meier curves of OS for patients categorised by HESI, OSI, HYSI, GSI, and ERSI in the GSE24080 dataset. (G) Circos plot representing the significant Spearman correlations across five cell stress indexes. OS, overall survival; HESI, heat stress index; OSI, oxidative stress index; HYSI, hypoxic stress index; GSI, genotoxic stress index; ERSI, endoplasmic reticulum stress index.

We performed univariate Cox analysis followed by LASSO regression to identify significant genes for constructing the following five models: heat stress index (HESI), oxidative stress index (OSI), hypoxic stress index (HYSI), genotoxic stress index (GSI) and endoplasmic reticulum stress index (ERSI) (Figure S2). LASSO regression analyses identified 4, 10, 9, 14 and 13 genes for building the HESI, OSI, HYSI, GSI and ERSI, respectively. The prognostic impact of all genes in five models is holistically illustrated through a forest plot using univariate Cox analysis in GSE24080 (Figure 1A). The median risk index was employed to stratify patients into high‐ and low‐risk in each model. Kaplan–Meier curves showed that the OS outcomes of high‐risk patients were significantly inferior to those of low‐risk patients within five stress models (all *p* < 0.001, Figure 1B–F). Spearman correlation analysis revealed that these five indexes were positively correlated with each other (Figure 1G).

### 3.2 Construction and evaluation of the CSI signature

**FIGURE 2:**
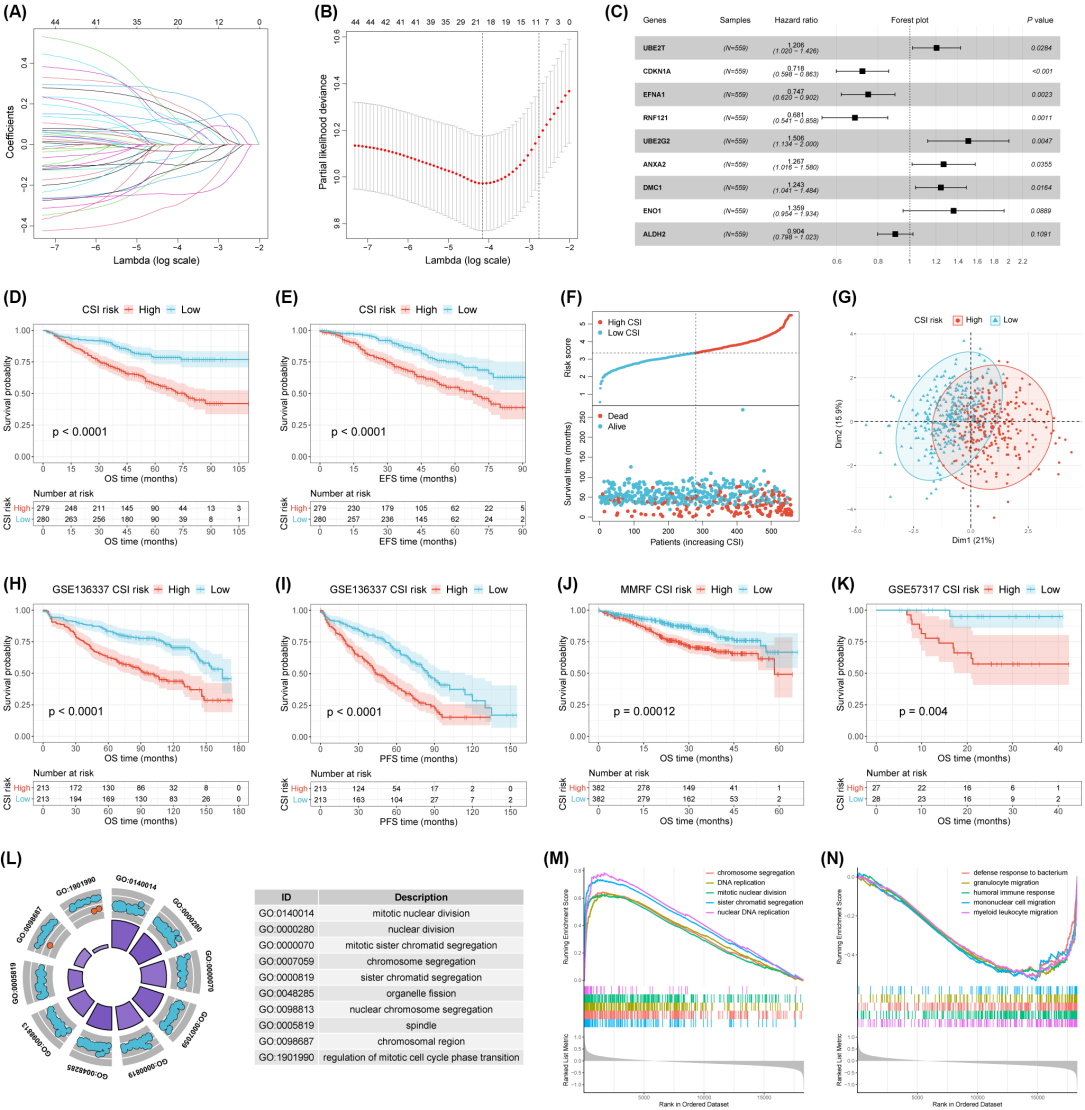
Establishment of an integrative CSI for MM patients. (A) LASSO coefficient profiles of 44 cell stress genes. (B) Cross‐validation for tuning parameter selection in the LASSO regression model. (C) Multivariate Cox regression analysis of the genes selected by LASSO regression analysis. (D, E) Kaplan–Meier curves of OS and EFS for patients grouped by CSI risk in the GSE24080 training set. (F) Distribution of risk score and survival status between CSI risk groups. (G) Principal Component Analysis (PCA) plot of high‐and low‐CSI groups. (H, I) Kaplan–Meier curves of OS and PFS for patients grouped by CSI risk in the GSE136337 dataset. (J, K) Kaplan–Meier curves of OS for patients grouped by CSI risk in MMRF‐COMMPASS and GSE57317 datasets. (L) Gene ontology (GO) circle plot of top 10 enriched biological functions between two CSI groups in the GSE24080 dataset. Blue represents upregulated genes and orange represents downregulated genes. (M, N) GSEA analysis for top enriched up‐ and down‐regulated biological functions in the training set. CSI, cellular stress index; EFS, event‐free survival; PFS, progression‐free survival.

To further establish a systemic model for cellular stress, we conducted LASSO regression analysis on 44 genes from the above five stress models and identified 21 candidates through 10‐fold cross‐validation (Figure 2A,B). After performing multivariate Cox stepwise regression, nine genes were eventually determined as robust markers to create the CSI signature (Figure 2C). The CSI of each MM patient was calculated using the following formula: CSI = (0.18734 × UBE2T expression) + (−0.33100 × CDKN1A expression) + (−0.29107 × EFNA1 expression) + (−0.38349 × RNF121 expression) + (0.40942 × UBE2G2 expression) + (0.23662 × ANXA2 expression) + (0.21746 × DMC1 expression) + (0.30648 × ENO1 expression) + (−0.10139 × ALDH2 expression). Patients were subsequently categorised into two risk groups according to the median CSI.

In the training cohort, Kaplan–Meier curves demonstrated that patients with high CSI had significantly inferior OS and event‐free survival (EFS) rates compared to those with low CSI (Figure 2D,E). High CSI was observed to be correlated with increasing mortality rates (Figure 2F). PCA analysis indicated a distinct cluster distribution between different CSI risk groups (Figure 2G). In the GSE136337 validation cohort, both OS and progression‐free survival (PFS) rates were notably lower in the high CSI group compared with the low CSI group (Figure 2H,I). In another two validation sets (MMRF‐COMMPASS and GSE57317), patients in the high CSI group showed poorer OS outcomes than those in the low CSI group (Figure 2J,K). Then, GO analysis revealed that CSI was involved in the enriched functions including mitotic nuclear division, sister chromosome segregation, organelle fission and spindle (Figure 2L). GSEA analyses confirmed the enrichment of these significant pathways in the high CSI group, such as DNA replication and chromosome segregation; while pathways activated in the low CSI group included defence response to bacterium, humoral immune response, and migration of granulocyte, mononuclear cell and myeloid leukocyte (Figure 2M,N).

### 3.3 Correlations between CSI and clinical features in MM patients

**FIGURE 3:**
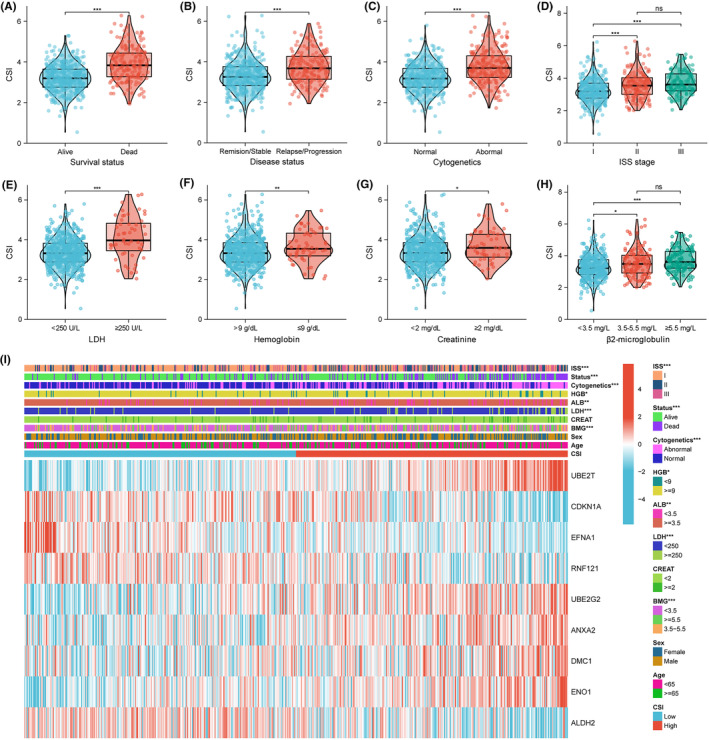
Relationships between CSI and clinical characteristics in MM. (A‐H) The associations of CSI with survival status, disease status, cytogenetic abnormalities, ISS stage, LDH, haemoglobin, creatinine, and β2‐microglobulin. (I) Heatmap of nine signature genes and clinical features between CSI subgroups. ALB, albumin; BMG, β2‐microglobulin; LDH, lactate dehydrogenase; HGB, haemoglobin; CREAT, creatinine; ISS, International Staging System. *p < 0.05, **p < 0.01, ***p < 0.001.

Thereafter, we investigated the associations of CSI with clinicopathological characteristics in the training cohort. CSI was significantly associated with survival and progression status of MM patients (Figure 3A,B). CSI levels were higher in patients with abnormal cytogenetics, ISS stage II or III, LDH ≥250 U/L, haemoglobin ≤9 g/dL, creatinine ≥2 mg/dL, and β2‐microglobulin ≥3.5 mg/L (Figure 3C–H). We further observed clear differences in the clinicopathological factors and gene expression between high and low CSI groups (Figure 3I).

### 3.4 Unsupervised consensus clustering of CSI genes

**FIGURE 4:**
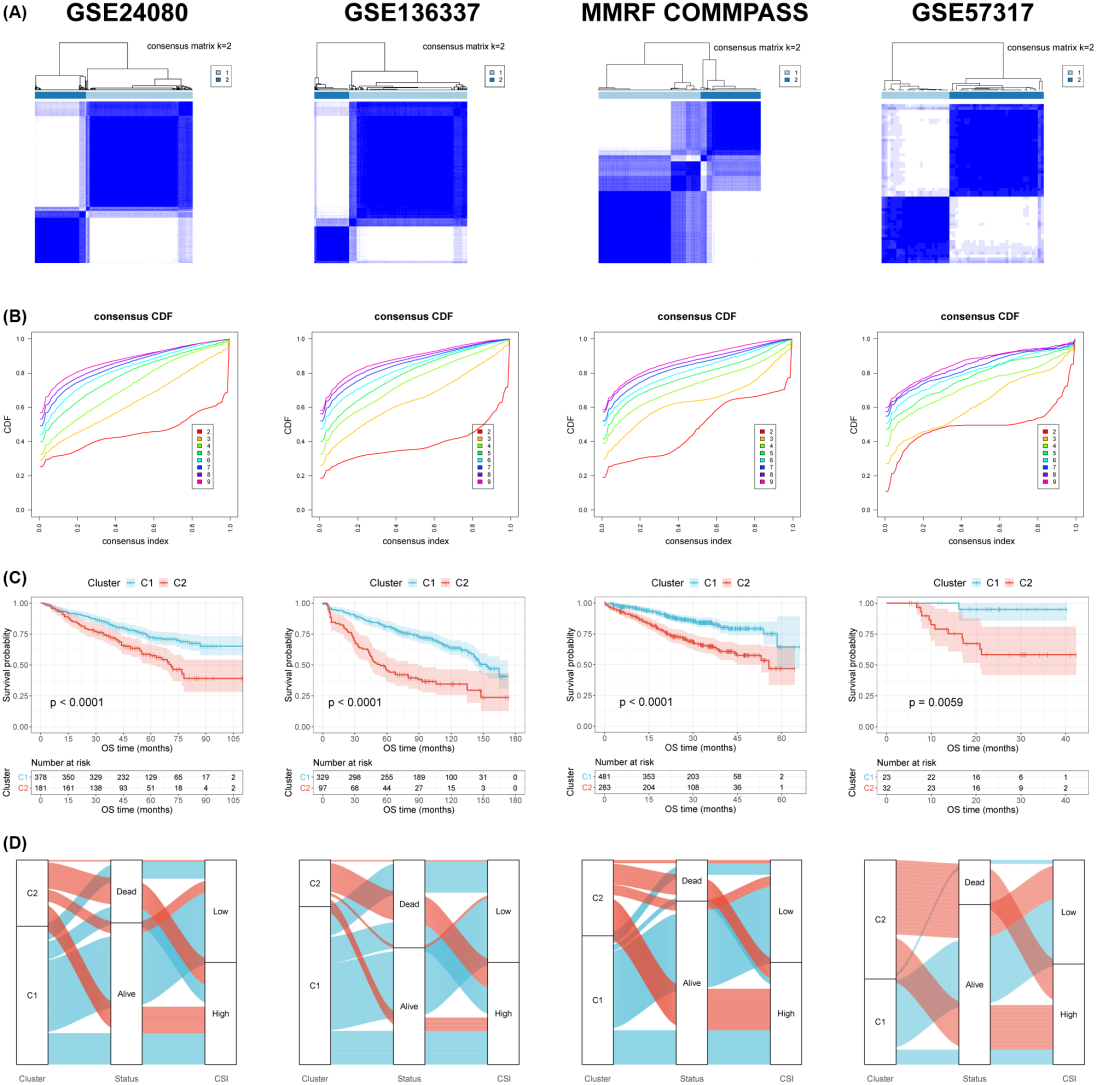
Identification of MM subtypes based on CSI genes using unsupervised clustering. (A) Consensus matrix heatmaps showing the clustering of MM samples according to CSI gene profiles at *k* = 2. (B) Cumulative distribution function curves displaying different clustering approaches for k values ranging from 2 to 9. (C) Kaplan–Meier survival analysis of the two distinct clusters of MM samples. (D) Sankey diagrams exhibiting the intercorrelations among molecular subtypes, survival status, and CSI risk in patients with MM.

By performing consensus clustering analyses of genes in the CSI model, we attempted to identify molecular subtypes of MM patients. After analysing four independent datasets (GSE24080, GSE136337, MMRF‐COMMPASS, and GSE57317), we found that the differences between subgroups were most prominent when k was equal to 2, indicating that MM samples could be effectively categorised into two clusters in each dataset (Figure 4A,B). The two subtypes differed markedly in terms of prognosis: C1 subtype had significantly better OS outcomes compared with those of C2 subtype (Figure 4C). Sankey plots revealed that the C1 subtype was predominantly linked to low CSI, while the C2 subtype was primarily associated with high CSI (Figure 4D).

### 3.5 Establishment and validation of a nomogram for OS prediction

**FIGURE 5:**
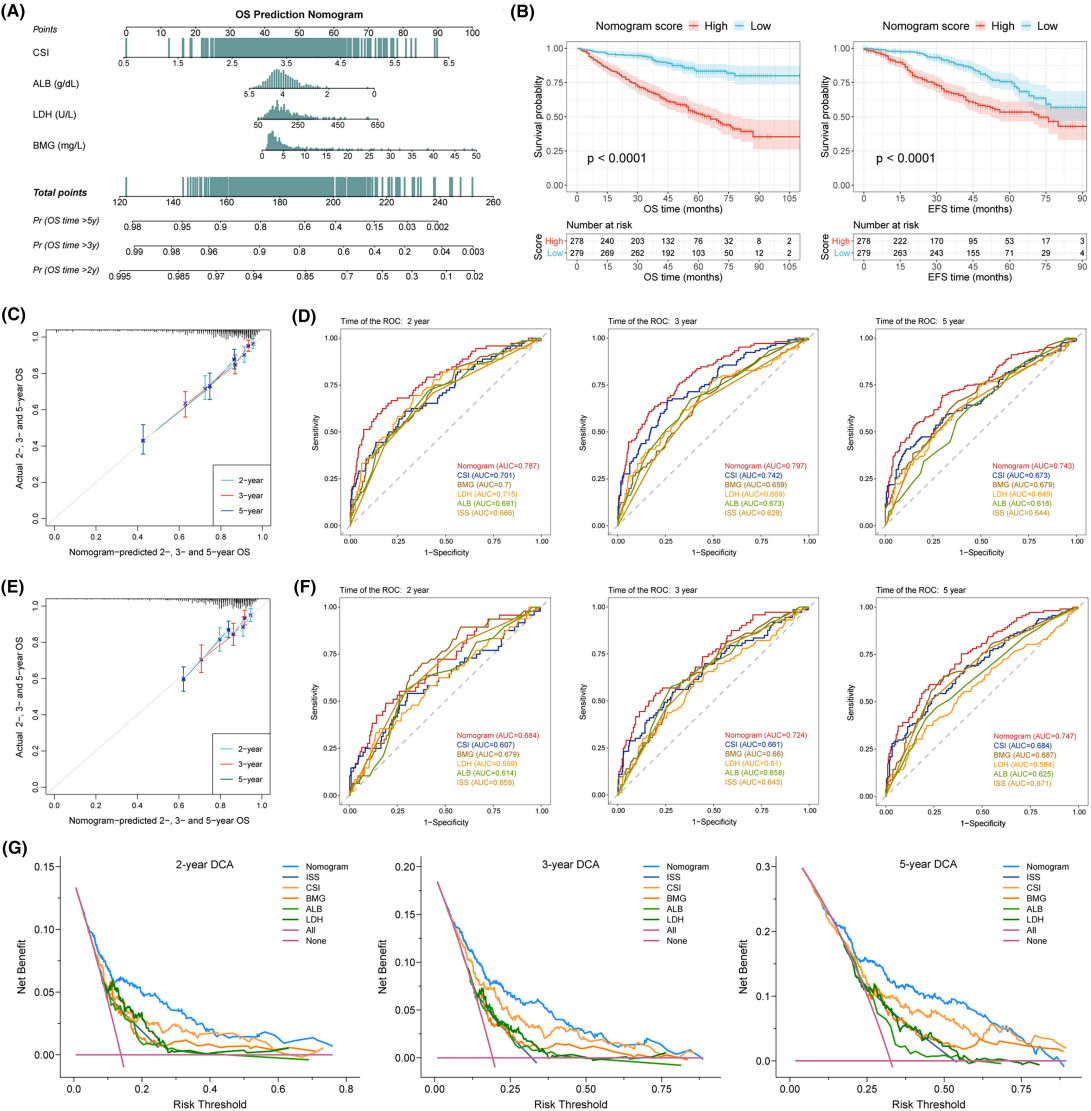
Development and validation of a prognostic nomogram model. (A) Nomogram for predicting 2‐, 3‐ and 5‐year OS probabilities. (B) Kaplan–Meier curves of OS and EFS for patients grouped by the nomogram score. (C) Calibration curves for predicting 2‐, 3‐ and 5‐year OS in the GSE24080 training cohort. (D) Receiver operating characteristic (ROC) curves of the nomogram and other features for 2‐, 3‐ and 5‐year OS prediction in the training cohort. (E) Calibration curves for predicting 2‐, 3‐ and 5‐year OS in the GSE136337 validation cohort. (F) ROC curves of the nomogram and other features for 2‐, 3‐ and 5‐year OS prediction in the validation cohort. (G) Decision curve analysis (DCA) of the nomogram and other features in predicting 2‐, 3‐ and 5‐year OS outcomes.

Univariate and multivariate Cox regression analyses were conducted to determine whether CSI was an independent prognostic indicator of OS (Table S2). Univariate analysis showed that high CSI was associated with inferior prognosis (HR: 2.718, *p* < 0.001), and multivariate analysis confirmed that CSI level could be an independent risk factor for OS (HR: 2.236, *p* < 0.001). We subsequently constructed a nomogram by incorporating significant variables identified by the multivariate Cox analysis, including LDH, ALB, BMG and CSI, to estimate 2‐, 3‐ and 5‐year OS probabilities (Figure 5A). Based on the total points calculated by our nomogram, there were pronounced disparities in OS and EFS rates between two groups with high and low nomogram scores (Figure 5B). Calibration curves demonstrated excellent agreement between nomogram prediction and actual reference (Figure 5C). The 2‐, 3‐ and 5‐year AUC of the nomogram were 0.787, 0.797 and 0.743, respectively, much higher than those of the other five features (Figure 5D). The nomogram showed consistent and robust performance in terms of accuracy and discrimination when validated in the GSE136337 cohort (Figure 5E,F). Additionally, DCA curves indicated that the nomogram exhibited superior clinical net benefits compared with other predictors, such as ISS stage (Figure 5G).

### 3.6 Analysis of tumour immune microenvironment

**FIGURE 6:**
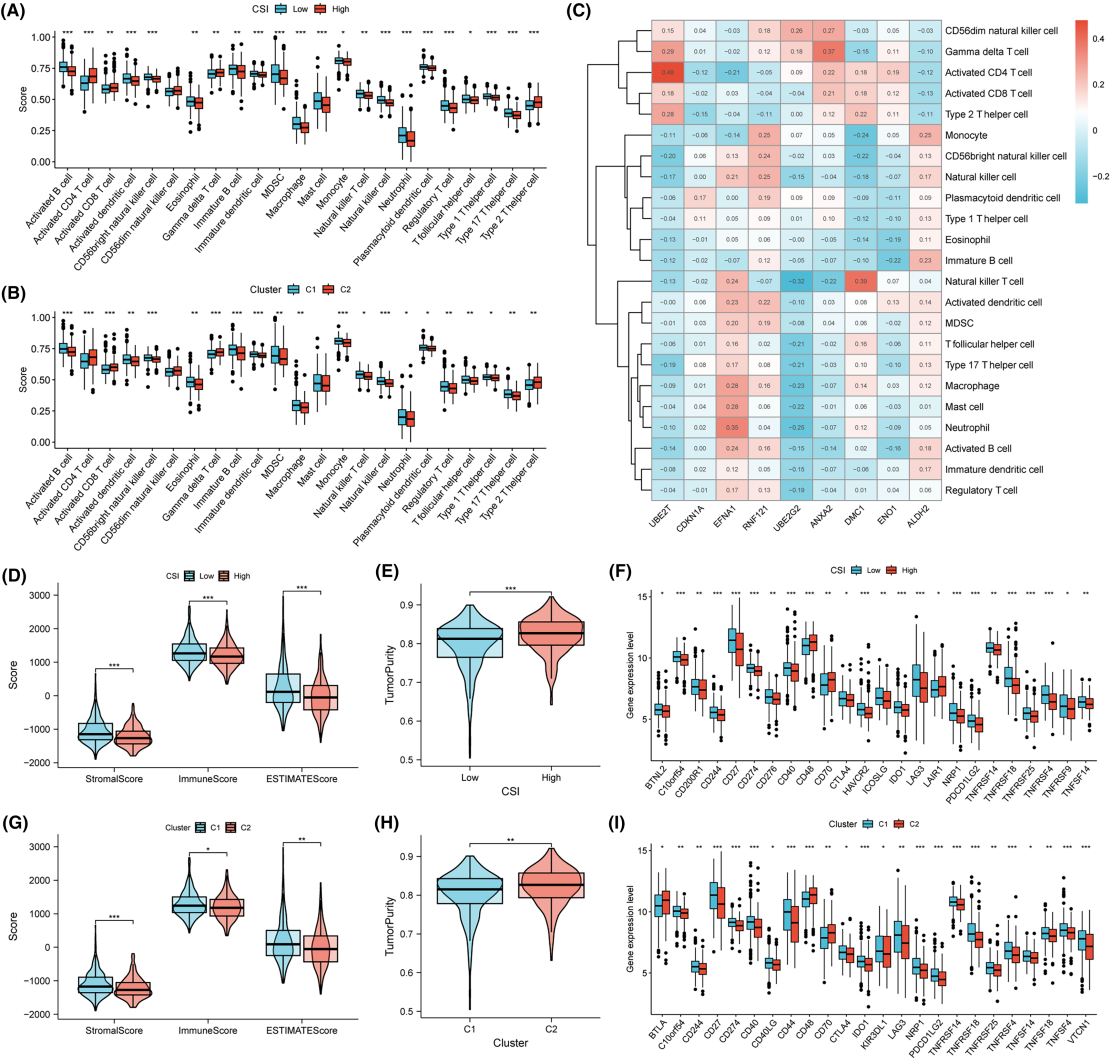
Characterisation of immune microenvironment based on the CSI signature. (A, B) Distribution of ssGSEA score in CSI risk groups and molecular clusters. (C) Heatmap showing the Spearman correlations between CSI genes and immune cell types. (D, E) Assessment of immune scores and tumour purity using ESTIMATE algorithm between CSI risk groups. (F) Expression patterns of immune checkpoint genes in CSI subgroups. (G, H) Assessment of immune scores and tumour purity using ESTIMATE algorithm between CSI molecular clusters. (I) Expression patterns of immune checkpoint genes in CSI clusters. ssGSEA, single sample gene set enrichment analysis; MDSC, myeloid‐derived suppressor cell. *p < 0.05, **p < 0.01, ***p < 0.001.

In general, the low CSI group or C1 cluster had a higher degree of immune cell infiltration, such as activated B cells, natural killer cells, macrophages, monocytes, regulatory T cells, and T helper 17 cells (Figure 6A,B). The heatmap depicted the correlations between infiltrated immune cells and each signature gene (Figure 6C). With respect to tumour microenvironment factors, the high CSI group possessed lower immune and stromal scores, but an increased estimation of tumour purity (Figure 6D,E). Expression levels of the majority of immune checkpoints, including CTLA4, LAG3 and PDCD1LG2, were found to be downregulated in the high CSI group (Figure 6F). Likewise, C2 subtype showed reduced immune and stromal cell abundance, higher tumour purity, and lower expression of immune checkpoints (Figure 6G–I).

### 3.7 Relationships between CSI and treatment response

**FIGURE 7:**
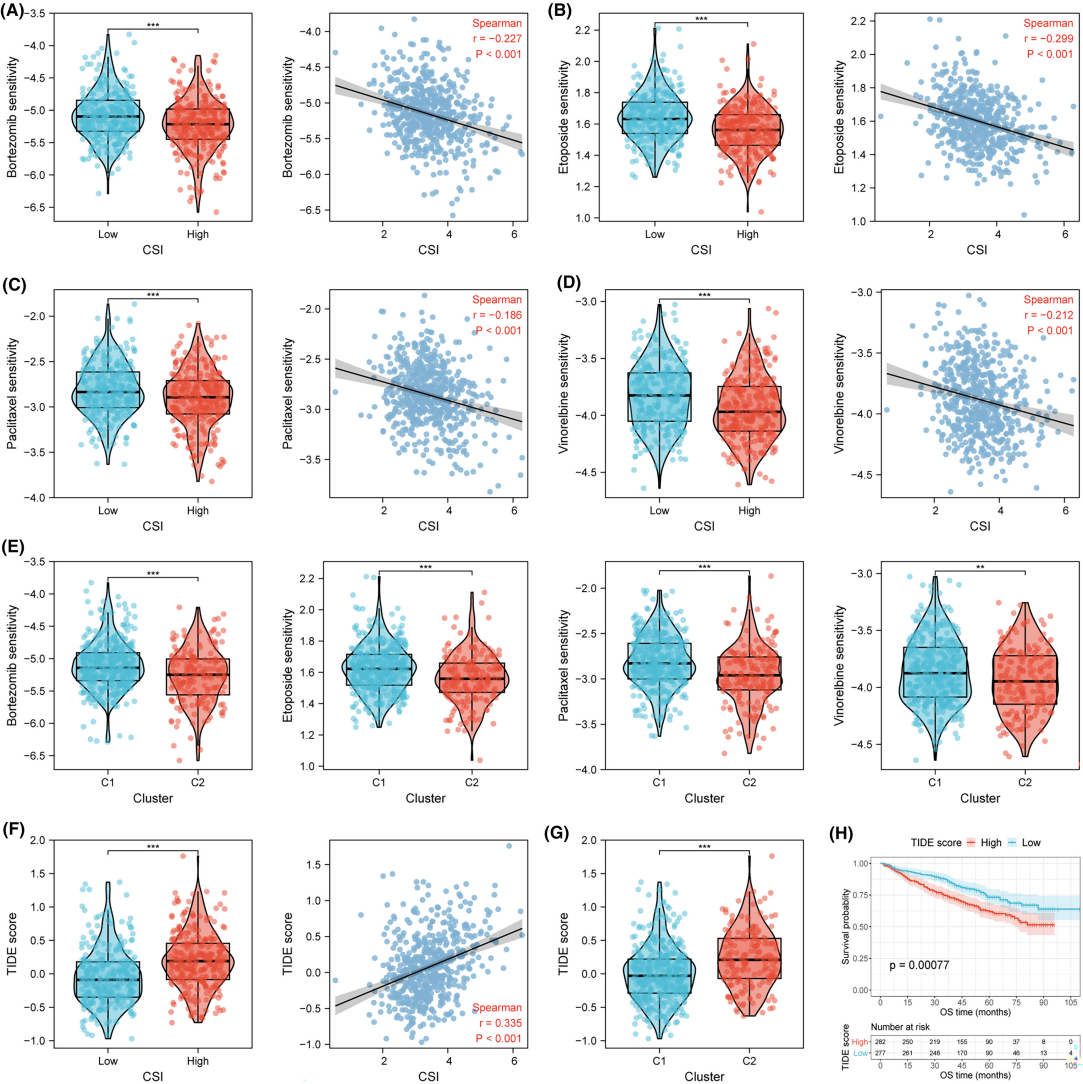
Correlation analysis of CSI in predicting chemotherapeutic sensitivity and immunotherapy response. (A‐D) Boxplots of drug sensitivity (bortezomib, etoposide, paclitaxel, and vinorelbine) between low‐ and high‐CSI groups, and correlation plots of CSI values and drug sensitivity in the GSE24080 cohort. (E) Boxplots of drug sensitivity (bortezomib, etoposide, paclitaxel, and vinorelbine) between molecular clusters. (F) Correlation of TIDE score with CSI values. (G) Comparison of TIDE score between CSI clusters. (H) Survival analysis between high and low TIDE score groups in MM patients. TIDE, Tumour Immune Dysfunction and Exclusion. **p < 0.01, ***p < 0.001.

To gain insight into the clinical significance of CSI, we performed sensitivity analyses on anti‐cancer drugs and immunotherapy. The IC50 values of bortezomib, etoposide, paclitaxel and vinorelbine were lower in the high CSI group, and significant negative correlations were observed between CSI and IC50 values of these drugs (all *p* < 0.001, Figure 7A–D). Patients with C2 subtype, much like those in the high CSI group, appeared to be more sensitive to the four common chemotherapeutic agents (Figure 7E). TIDE score was calculated for each patient to assess the immune evasion capacity of MM cells. Patients in the high CSI group or C2 subtype had a higher TIDE score, and there was a significant positive correlation between CSI and TIDE score (all *p* < 0.001, Figure 7F,G). Moreover, high TIDE score was associated with a worse prognosis in MM patients (*p* < 0.001, Figure 7H). These observations suggest that patients with low CSI are more likely to be sensitive to immune checkpoint blockade (ICB) treatment.

### 3.8 Expression patterns and external validation of CSI genes

**FIGURE 8:**
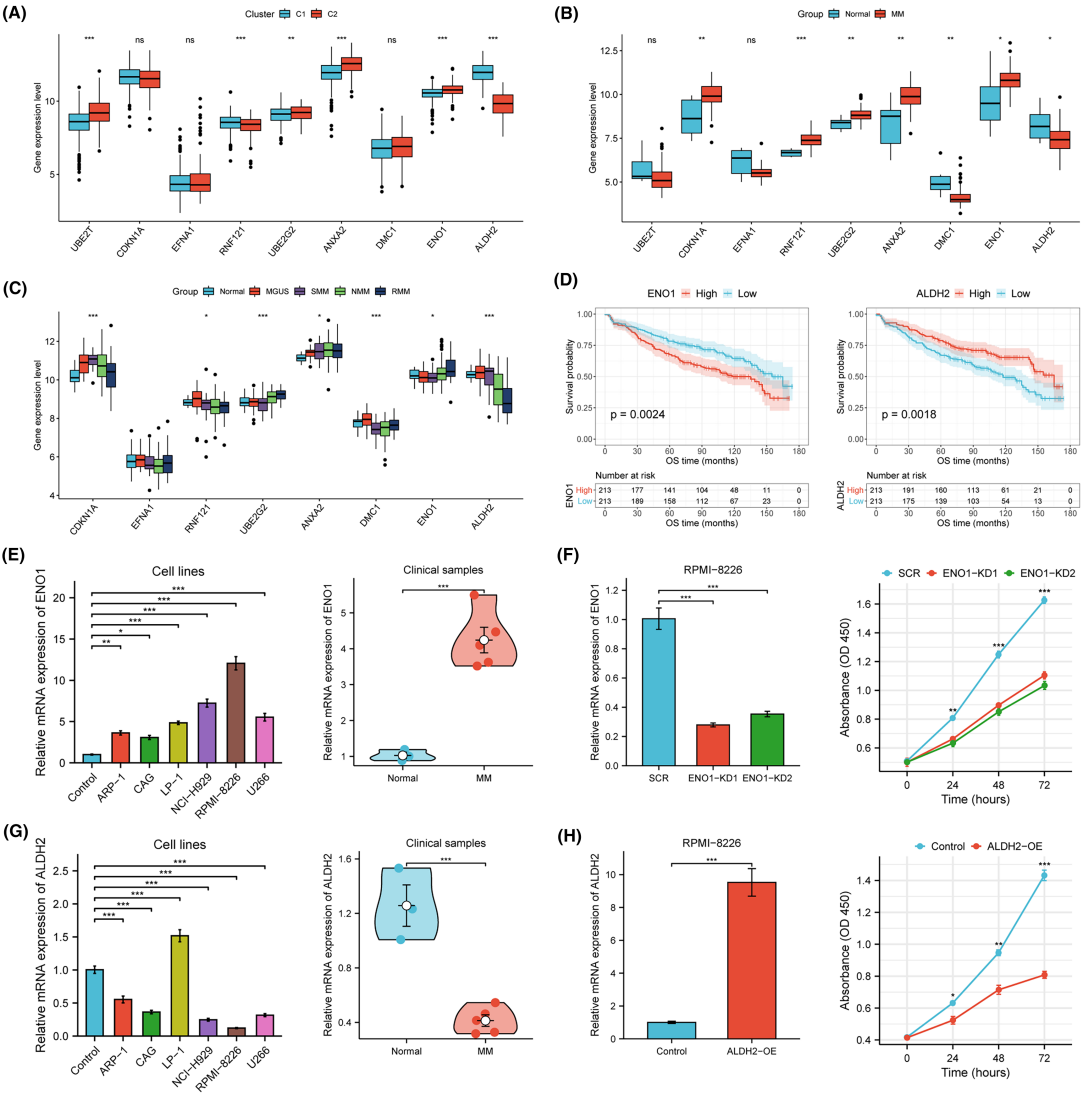
Expression profiles of the CSI signature and experimental validation of ENO1 and ALDH2. (A) Expression patterns of nine model genes between CSI clusters in the GSE24080 dataset. (B) Expression differences of CSI genes in normal and MM samples in the GSE39754 dataset. (C) Expression levels of CSI genes during different stages of MM progression in the GSE6477 dataset (UBE2T not available). (D) Survival curves showing the prognostic effects of ENO1 and ALDH2 in MM. (E) Validation of ENO1 expression in cell lines and clinical samples. (F) qPCR detection and CCK‐8 assay of ENO1‐knockdown RPMI‐8226 cells. (G) Validation of ALDH2 expression in cell lines and clinical samples. (H) qPCR detection and CCK‐8 assay of ALDH2‐overexpression RPMI‐8226 cells. MGUS, monoclonal gammopathy of undetermined significance; SMM, smouldering multiple myeloma; NMM, newly diagnosed multiple myeloma; RMM, relapsed multiple myeloma. *p < 0.05, **p < 0.01, ***p < 0.001.

We examined the expression levels of genes in the CSI signature under different scenarios. Differential expression was observed in UBE2T, RNF121, UBE2G2, ANXA2, ENO1 and ALDH2 between C1 and C2 subtypes (Figure 8A). Compared with healthy donors, CDKN1A, RNF121, UBE2G2, ANXA2 and ENO1 were upregulated in MM patients, whereas DMC1 and ALDH2 were both downregulated (Figure 8B). UBE2G2 and ENO1 expression increased from SMM to NMM and RMM, while ALDH2 showed the opposite trend (Figure 8C). Considering the expression and prognostic characteristics of these candidates, we selected ENO1 as a risk gene and ALDH2 as a suppressor gene for further verification. In the GSE136337 validation cohort, high ENO1 expression and low ALDH2 expression were correlated with poor prognosis, respectively (Figure 8D).

Further validation was performed using qPCR in MM cell lines and patient specimens. ENO1 expression was strikingly upregulated in all six MM cell lines and clinical samples compared with normal controls (Figure 8E). We silenced ENO1 in the RPMI‐8226 cell line that showed the highest expression of ENO1, and observed a significant inhibition of cellular proliferation (Figure 8F). By contrast, ALDH2 exhibited low expression in these MM cell lines (except LP‐1) and among MM patients (Figure 8G). We therefore overexpressed ALDH2 in RPMI‐8226 cells that happened to have the lowest ALDH2 expression, and found that ALDH2‐upregulated cells were significantly less proliferative (Figure 8H).

## 4 DISCUSSION

The role of cellular stress in the pathogenesis and progression of MM is fundamental. MM cells are exposed to various forms of cellular stress, and the preservation of specific stress‐response pathways is crucial for ensuring their survival. Targeting these pathways or molecules presents a promising avenue for treating MM.^24^ However, the impact of cell stress‐related genes on MM prognosis and treatment remains inadequately supported by the available evidence. Here, for the first time, we created a novel cellular stress‐related prognostic index to assess clinical outcomes in MM. The CSI signature can function as a pragmatic tool for discerning common clinical features and directing individualised therapeutic strategies. We also conducted in vitro experiments, demonstrating the differential expression of ENO1 and ALDH2 in MM and their ability to regulate the proliferation of MM cells.

We initially developed five distinct cellular stress indexes that were highly interrelated and significantly predictive of patient outcomes. Further, we integrated the five indexes to construct a CSI model with an enhanced predictive capability, which was well validated in independent external datasets. High CSI is closely associated with clinical indicators for dismal outcomes and can effectively differentiate between different stages and disease status. Two molecular subtypes of MM were identified based on CSI genes, with the C2 subtype exhibiting similar biological and clinical characteristics to those observed in high CSI populations. Given the significant prognostic impact of CSI in MM patients, we sought to develop a nomogram model incorporating CSI and clinical features for more accurate prediction of survival outcomes. Multivariate analysis substantiated the independent prognostic role of CSI. The nomogram demonstrated that CSI was the most significant predictor of OS compared with nearly all other factors. Through internal and external validation, the predictive accuracy and discriminability of our nomogram were well confirmed, which is expected to inform decision‐making in clinical practise. Compared to similar prediction models in MM,^25^, ^26^, ^27^ our model offers several advantages including larger sample sizes for modelling and validation, more robust modelling methods and higher nomogram AUC values. Although the prognostic value of CSI is apparent, further verification through large‐scale multicenter studies is necessary.

Meanwhile, we conducted functional enrichment and immune status analyses to elucidate the underlying biological mechanisms through which CSI influences patient prognosis, as well as to determine optimal treatment regimens. In the high CSI group, upregulated genes were mainly enriched in cell division events, such as mitotic nuclear division, chromosome segregation, and DNA replication. This implies that patients with high CSI may experience a worse prognosis due to an elevated level of cellular proliferation. Coincidentally, high CSI patients exhibit greater sensitivity to antimitotic agents like paclitaxel and vinorelbine, thereby advocating for the combination of existing therapies with antimitotic drugs to improve therapeutic efficacy for such patients. Bortezomib or etoposide treatment is also likely to confer more survival benefits to patients with higher CSI. On the other hand, enriched downregulated pathways in high CSI samples are largely related to immune responses and immune cell migration. As anticipated, the high CSI risk group showed reduced levels of immune cell infiltration and immune score, indicative of compromised immune functions.^28^ A high CSI is also correlated with an enhanced capacity of immune escape, resulting in tumour persistence and worse prognosis.^29^ Conversely, patients with low CSI had enhanced immune checkpoint expression and lower TIDE scores, suggesting that these patients may be more responsive to ICB immunotherapy.^23^, ^30^ Hence, it is recommended to choose suitable treatment protocols based on the level of CSI.

Our CSI model incorporates a total of nine prognostic genes, comprising five risk factors (UBE2T, UBE2G2, ANXA2, DMC1 and ENO1) and four protective factors (CDKN1A, EFNA1, RNF121 and ALDH2). These genes are implicated in typical stress regulatory mechanisms and have a close association with tumour biology. UBE2T confers chemotherapy resistance and promotes tumour growth by alleviating DNA replication stress.^31^, ^32^ Upregulation of UBE2T expression is considered as a possible driver of MM initiation and aggressiveness.^33^ Amplified and overexpressed UBE2T is essential for the activity of homologous recombination in MM, and UBE2T‐deficient MM cells become more sensitive to DNA damaging agents.^34^ Our result is congruent with previous findings that increased expression of UBE2T is correlated with a poor prognosis in MM.^35^, ^36^ CDKN1A (P21) is a cyclin‐dependent kinase (CDK) inhibitor that suppresses MM cell proliferation.^37^, ^38^ We validated its role as a tumour suppressor through prognostic bioinformatics analysis. EFNA1 is highly induced under hypoxic stress through HIF‐dependent pathways in cancer cells, and elevated EFNA1 expression is generally linked with an unfavourable prognosis in solid tumours.^39^ RNF‐121 is an ER‐anchored ubiquitin ligase located downstream of the PERK‐mediated UPR pathway, and its expression can be induced through the UPR activation.^40^, ^41^ UBE2G2 is a ubiquitin‐conjugating enzyme that degrades numerous misfolded ER proteins.^42^ More evidence is required to confirm the prognostic value of EFNA1, RNF121 and UBE2G2 in MM. ANXA2 is reported to be significantly upregulated and correlate with inferior survival outcomes in MM,^43^ which is consistent with our findings. ANXA2 promotes proliferation, inhibits apoptosis, and enhances angiogenesis in MM cells.^44^ Thus far, an ANXA2 binding aptamer wh6 has been screened and characterised to successfully block MM cell proliferation induced by ANXA2.^45^

ENO1 is a multifunctional glycolytic enzyme and oncoprotein that exists as a plasminogen receptor on the cell surface and is involved in most of the ‘cancer hallmarks’. It participates in cellular stress, accelerates various cancer‐promoting events and helps tumours escape immune destruction. ENO1 is frequently upregulated in various tumour tissues and often associates with unfavourable prognostic outcomes.^46^, ^47^ Preclinical experiments have suggested that immunotherapy targeting ENO1 in MM can restore anti‐tumour immunity and enhance tumour‐killing activities.^48^ We confirmed the expression level and prognostic effect of ENO1 in MM, and found that it promotes the proliferative phenotype of MM cells. The results provide a clear direction that targeting ENO1 is an effective strategy for treating MM. In contrast, our findings indicate that ALDH2 exerts a tumour suppressive effect, as evidenced by its downregulation in MM patients and overexpression‐induced inhibition of MM cells. ALDH2 deficiency can contribute to tumorigenesis through mechanisms such as autophagy dysregulation, epigenetic instability, and immune system dysfunction.^49^ Notably, single nucleotide polymorphisms (SNPs) in ALDH2 were strongly associated with the response to melphalan and OS outcomes in MM patients treated with high‐dose melphalan.^50^ Nevertheless, the exact functions of these CSI genes in MM remain poorly understood and necessitate further in vivo validation and detailed mechanistic investigations.

## 5 CONCLUSION

To conclude, we constructed and verified an integrative CSI for prognostic prediction in MM by selecting key genes from five different types of cellular stress. Different characteristics were observed in the clinical prognosis, immune microenvironment, and treatment response between CSI subgroups or clusters. A nomogram survival model incorporating CSI was built for precise risk stratification, and the nomogram demonstrated good performance in terms of discrimination, calibration, and clinical utility. The CSI proposed in this study holds promise to become a reliable predictor for clinical outcomes and drug sensitivity in MM.

## AUTHOR CONTRIBUTIONS

**Jiaxuan Xu:** Formal analysis (lead); visualization (lead); writing – original draft (lead). **Xiaoqing Dong:** Validation (equal). **Jiahui Dong:** Resources (equal). **Yue Peng:** Data curation (equal). **Mengying Xing:** Software (equal). **Lanxin Chen:** Methodology (equal). **Quan Zhao:** Project administration (equal). **Bing Chen:** Project administration (equal).

## FUNDING INFORMATION

This work was supported by the National Natural Science Foundation of China (82273954, 82304987).

## CONFLICT OF INTEREST STATEMENT

The authors declare no conflicts of interest related to this study.

## Supporting information


Data S1.

## Data Availability

The data sets in this research can be obtained from the corresponding authors on reasonable request.
