# Supplementary material for: Leveraging diverse cellular stress patterns for predicting clinical outcomes and therapeutic responses in patients with multiple myeloma
Source: J Cell Mol Med. 2024 Sep 8;28(17):e70054. doi: 10.1111/jcmm.70054 (PMC11381192; doi:10.1111/jcmm.70054)
Supplement: Supplementary file 1 — Data S1. [file JCMM-28-e70054-s001.pdf]

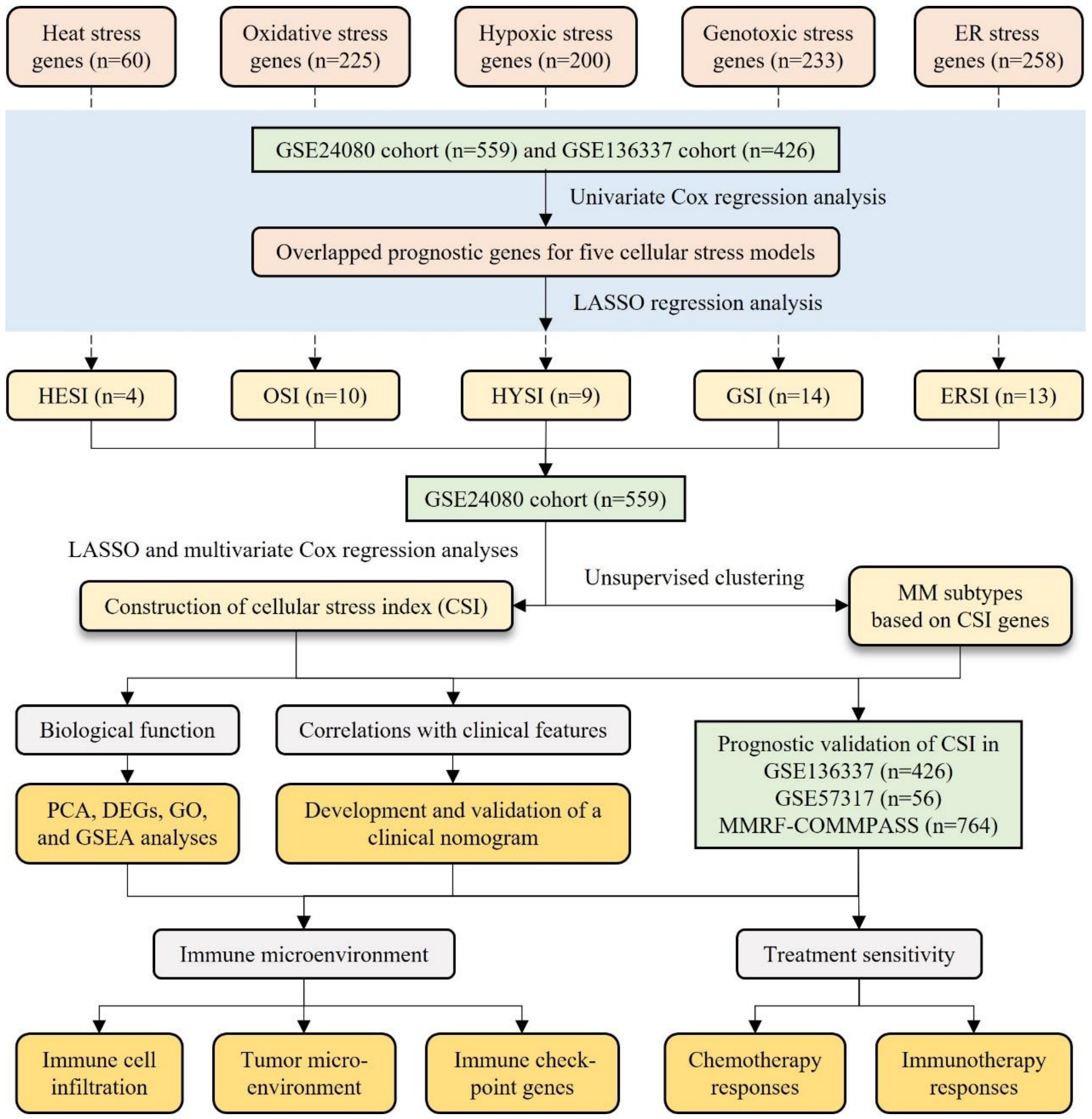

Figure S1. A flow diagram of analytical methods in this study

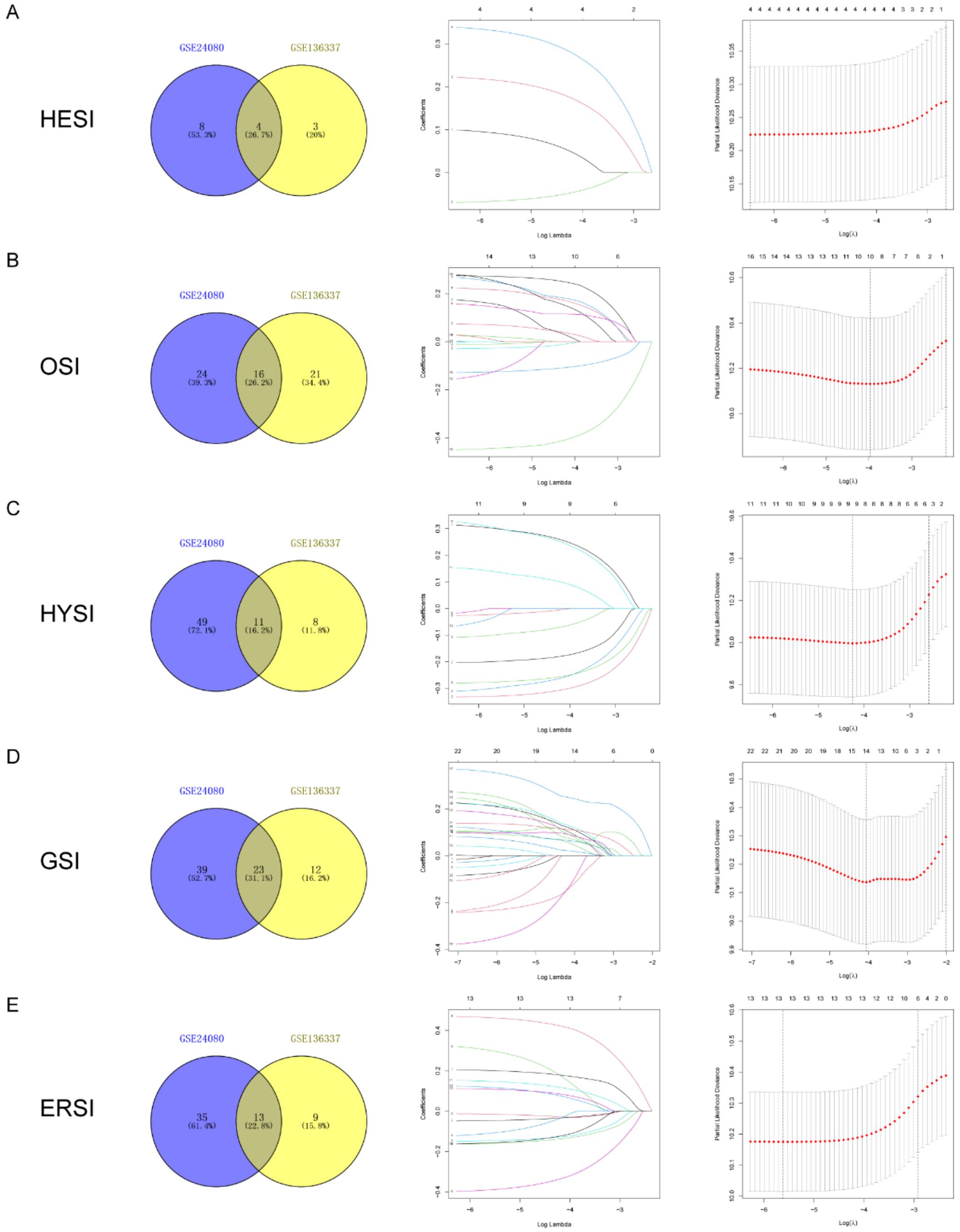

Figure S2. Construction of five cell stress-related prognostic indexes. (A-F) From left to right: Venn diagrams of significant prognosis-associated and cell stress-related genes in MM; LASSO regression coefficients of cell stress-related genes; 10-fold cross-validation for selecting the optimal parameters in LASSO models. HESI, heat stress index; OSI, oxidative stress index; HYSI, hypoxic stress index; GSI, genotoxic stress index; ERSI, endoplasmic reticulum stress index.

Table S1. Primer sequences for qRT-PCR analysis

Gene symbol	Direction	Sequence information (5'-3')
ENO1	Forward	TGCGTCCACTGGCATCTAC
	Reverse	CAGAGCAGGCGCAATAGTTTTA
ALDH2	Forward	TGTGTGGGTCAACTGCTATGA
	Reverse	TCACTTCAGTGTATGCCTGCA
GAPDH	Forward	GAAGGTGAAGGTCGGAG
	Reverse	GAAGATGGTGATGGGATTTC

Table S2. Univariate and multivariate Cox regression analysis of OS in the GSE24080 training cohort

**Characteristics**	**Univariate analysis**			**Multivariate analysis**		
	**HR**	**95% CI**	***P* value**	**HR**	**95% CI**	***P* value**
Age (≥65 vs. <65 years)	1.216	0.871-1.697	0.252			
Sex (male vs. female)	0.971	0.716-1.317	0.850			
BMPC	1.010	1.004-1.016	<0.001	1.002	0.995-1.009	0.558
BMG	1.084	1.066-1.103	<0.001	1.116	1.069-1.165	<0.001
CREAT	1.240	1.146-1.342	<0.001	0.874	0.755-1.011	0.070
LDH	1.006	1.005-1.008	<0.001	1.003	1.001-1.005	0.001
ALB	0.569	0.463-0.700	<0.001	0.722	0.563-0.924	0.010
HGB	0.867	0.799-0.940	<0.001	1.051	0.945-1.169	0.359
CRP	1.004	0.999-1.008	0.131			
ISS stage (II vs. I)	1.606	1.099-2.347	0.014	0.937	0.605-1.452	0.771
ISS stage (III vs. I)	2.954	2.084-4.189	<0.001	0.767	0.431-1.366	0.368
CSI	2.718	2.244-3.293	<0.001	2.236	1.801-2.776	<0.001

OS, overall survival; HR, hazard ratio; CI, confidence interval; BMPC, bone marrow plasma cells; BMG, β2-microglobulin; CREAT, creatinine; LDH, lactate dehydrogenase; ALB, albumin; HGB, hemoglobin; CRP, C-reactive protein; ISS, International Staging System; CSI, cellular stress index.
